# Induction of apoptosis in myeloid leukaemic cells by ribozymes targeted against AML1/MTG8

**DOI:** 10.1038/sj.bjc.6690214

**Published:** 1999-03

**Authors:** H Matsushita, M Kizaki, H Kobayashi, A Muto, Y Ikeda

**Affiliations:** Division of Haematology, Keio University School of Medicine, Tokyo, Japan; Department of Laboratory Medicine, National Defense Medical College, Saitama, Japan

**Keywords:** apoptosis, ribozyme, AML, AML1/MTG8, t(8;21)

## Abstract

The translocation (8;21)(q22;q22) is a karyotypic abnormality detected in acute myeloid leukaemia (AML) M2 and results in the formation of the chimeric fusion gene *AML1/MTG8*. We previously reported that two hammerhead ribozymes against *AML1/MTG8* cleave this fusion transcript and also inhibit the proliferation of myeloid leukaemia cell line Kasumi-1 which possesses t(8;21)(q22;q22). In this study, we investigated the mechanisms of inhibition of proliferation in myeloid leukaemic cells with t(8;21)(q22;q22) by ribozymes. These ribozymes specifically inhibited the growth of Kasumi-1 cells, but did not affect the leukaemic cells without t(8;21)(q22;q22). We observed the morphological changes including chromatin condensation, fragmentation and the formation of apoptotic bodies in Kasumi-1 cells incubated with ribozymes for 7 days. In addition, DNA ladder formation was also detected after incubation with ribozymes which suggested the induction of apoptosis in Kasumi-1 cells by the *AML1/MTG8* ribozymes. However, the ribozymes did not induce the expression of CD11b and CD14 antigens in Kasumi-1 cells. The above data suggest that these ribozymes therefore inhibit the growth of myeloid leukaemic cells with t(8;21)(q22;q22) by the induction of apoptosis, but not differentiation. We conclude therefore that the ribozymes targeted against *AML1/MTG8* may have therapeutic potential for patients with AML carrying t(8;21)(q22;q22) while, in addition, the product of the chimeric gene is responsible for the pathogenesis of myeloid leukaemia. © 1999 Cancer Research Campaign


*British Journal of Cancer* (1999) **79**(9/10), 1325–1331
© 1999 Cancer Research Campaign
Article no. bjoc.1998.0214

### Induction of apoptosis in myeloid leukaemic cells by ribozymes targeted against AML1/MTG8

**H Matsushita[1], M Kizaki[1], H Kobayashi[2], A Muto[1] and Y Ikeda[1]**

[1]Division of Haematology, Keio University School of Medicine, Tokyo; and [2]Department of Laboratory Medicine, National Defense Medical College, Saitama, Japan

**Summary** The translocation (8;21)(q22;q22) is a karyotypic abnormality detected in acute myeloid leukaemia (AML) M2 and results in the formation of the chimeric fusion gene *AML1/MTG8*. We previously reported that two hammerhead ribozymes against *AML1/MTG8* cleave this fusion transcript and also inhibit the proliferation of myeloid leukaemia cell line Kasumi-1 which possesses t(8;21)(q22;q22). In this study, we investigated the mechanisms of inhibition of proliferation in myeloid leukaemic cells with t(8;21)(q22;q22) by ribozymes. These ribozymes specifically inhibited the growth of Kasumi-1 cells, but did not affect the leukaemic cells without t(8;21)(q22;q22). We observed the morphological changes including chromatin condensation, fragmentation and the formation of apoptotic bodies in Kasumi-1 cells incubated with ribozymes for 7 days. In addition, DNA ladder formation was also detected after incubation with ribozymes which suggested the induction of apoptosis in Kasumi-1 cells by the *AML1/MTG8* ribozymes. However, the ribozymes did not induce the expression of CD11b and CD14 antigens in Kasumi-1 cells. The above data suggest that these ribozymes therefore inhibit the growth of myeloid leukaemic cells with t(8;21)(q22;q22) by the induction of apoptosis, but not differentiation. We conclude therefore that the ribozymes targeted against *AML1/MTG8* may have therapeutic potential for patients with AML carrying t(8;21)(q22;q22) while, in addition, the product of the chimeric gene is responsible for the pathogenesis of myeloid leukaemia.

**Keywords:** apoptosis; ribozyme; AML; AML1/MTG8; t(8;21)

The t(8;21)(q22;q22) translocation is one of the specific chromosome translocations which exists in 7–17% of the cases with acute myeloid leukaemia (AML) and is usually classified as AML M2 according to the French–American–British (FAB) classification criteria (Koeffler, 1987; Schiffer et al, 1989; Tashiro et al, 1992). This translocation results in the formation of the *AML1/MTG8* chimeric fusion gene, which has a 5′ portion of the *AML1* gene on chromosome 21 which is fused almost to the entire *MTG8* gene, also called *ETO* gene, on chromosome 8 (Erickson et al, 1992; Miyoshi et al, 1993).

The AML1 is a member of transcriptional factors which contain a region of homology to the Drosophila pair-rule gene, *runt* (Miyoshi et al, 1993). The runt homology domain is responsible for heterodimerization with core binding factor β (CBFβ) (Lenny et al, 1995) and specific binding to the sequence TGT/cGGT which is the enhancer core motif (Meyers et al, 1993). Several target genes for AML1 have been identified such as neutrophil elastase, myeloperoxidase, GM-CSF, IL-3, M-CSF receptor and TCRβ enhancer (Cameron et al, 1994; Nuchprayoon et al, 1994; Zhang et al, 1994, 1996; Frank et al, 1995; Meyers et al, 1995; Takahashi et al, 1995). AML1 has been found to transactivate the regulatory segments of each of these genes. Three representative forms of proteins, AML1a, AML1b and AML1c, are produced from the *AML1* gene by alternative splicing (Miyoshi et al, 1995). AML1a and AML1b are thought to regulate haematopoietic myeloid cell differentiation and transcriptional activation antagonistically (Tanaka et al, 1995). The function of the AML1 protein is regulated by extracellular signal-regulated kinase (ERK), a member of the mitogen-activated protein kinases through phosphorylation (Tanaka et al, 1996). An in vivo study revealed that mice lacking *AML1* are embryonically lethal without myeloid or erythroid progenitors originating from definitive haematopoiesis (Okuda et al, 1996; Wang et al, 1996). These data indicate that AML1 is one of the essential transcriptional factors for the definitive haematopoiesis of all types of cell lineage.

AML1/MTG8 chimeric protein shares structural features with these genes and also demonstrates the runt homology domain (Miyoshi et al, 1993). *AML1/MTG8* is thought to interfere with AML1b-dependent transcriptional activation as a dominant negative protein (Meyers et al, 1993, 1995). The antisense oligonucleotide targeted against *AML1/MTG8* fusion transcript inhibited the growth of the AML cell lines with t(8;21)(q22;q22) (Sakakura et al, 1994). Therefore, AML1/MTG8 chimeric fusion protein is considered to play an important role in the leukaemogenesis of acute myeloid leukaemia with t(8;21)(q22;q22).

Hammerhead ribozymes are one of the useful tools for the specific inhibition of gene expression (Haseloff and Gerlach, 1988). They are oligoribonucleotides with sequence-specific cleavage activity of target RNA and can be designed to cleave any triplet of NUX (N = any nucleotide, X = A, C or U) (Koizumi et al, 1988). One molecule of ribozyme can cleave a plural number of target RNAs by repeating the catalytic cycle. Therefore ribozymes are thought to be more effective than antisense oligonucleotides to suppress the expression of the target genes (Homann et al, 1993).

We have designed two hammerhead ribozymes against

*Received 12 November 1997*
*Revised 13 August 1998*
*Accepted 8 October 1998*

*Correspondence to:* M Kizaki

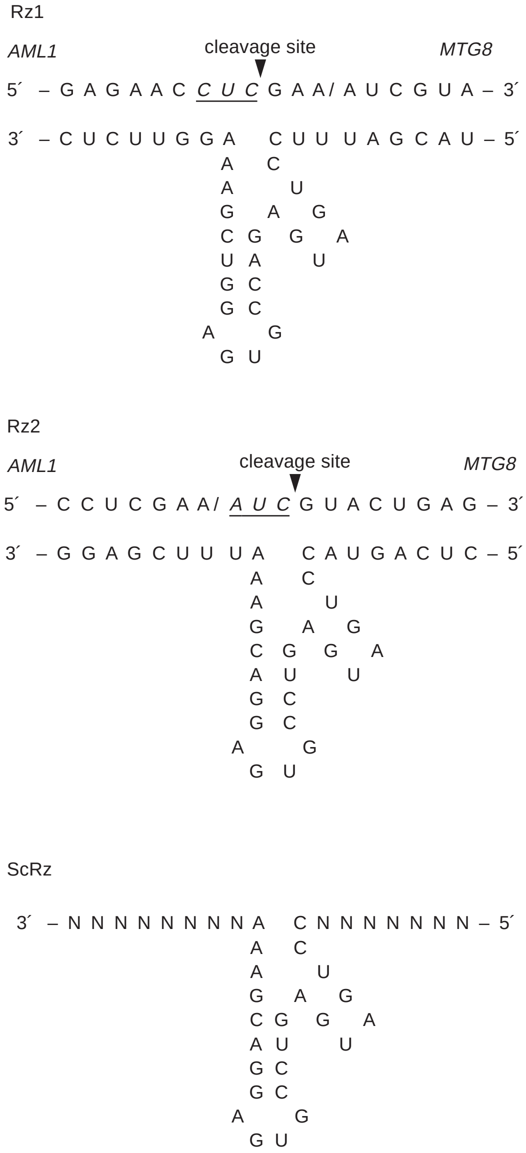

**Figure 1** Structures of two hammerhead ribozymes Rz1 and Rz2 targeted against *AML1/MTG8*, and unrelated scramble ribozyme (ScRz). These hammerhead ribozymes were designed based on the model of Haseloff and Gerlach (1988). Rz1 cleaves the CUC sequence located three bases upstream from the break point and Rz2 cleaves the AUC sequence located three bases downstream from the breakpoint. These two hammerhead ribozymes specifically cleaved the *AML1/MTG8* substrate in a cell-free system, as expected (Matsushita et al, 1995). ScRz has random nucleotides in 3′- and 5′-complementary arms, and thus it was not able to cleave the *AML1/MTG8* substrate. 'N' means any nucleotides

*AML1/MTG8* which specifically cleaved the *AML1/MTG8* substrate in a cell-free system and also inhibited the growth of Kasumi-1 cells, an AML cell line with t(8;21)(q22;q22) (Matsushita et al, 1995). The purpose of this report is to disclose the mechanism of growth inhibition of AML cells with t(8;21)(q22;q22) by these ribozymes. We herein demonstrate for the first time that ribozymes can inhibit the proliferation of myeloid leukaemic cells by the induction of apoptosis, but not differentiation.

### MATERIALS AND METHODS

### Cells and chemicals

The myeloid leukaemic cell line Kasumi-1 established from a patient with AML M2 carrying t(8;21)(q22;q22) was a generous gift of Dr N Kamada (Hiroshima University, Horoshima, Japan) (Asou et al, 1991). The other human myeloid leukaemic cell lines used in this study were HL-60, KG-1, NB4 (a gift from Dr M Lanotte, Hôpital St. Louis, Paris, France) (Lanotte et al, 1991) and

 *© Cancer Research Campaign 1999*

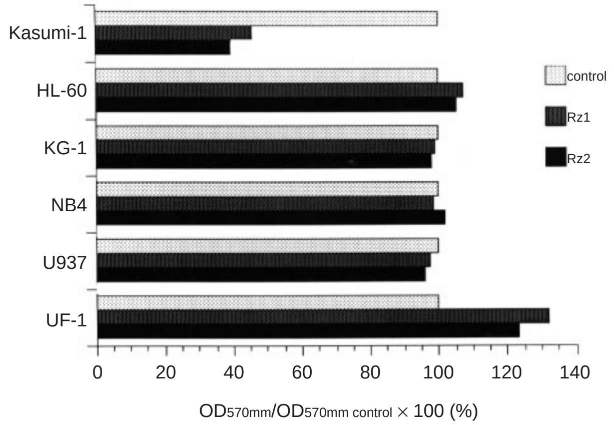

**Figure 2** Inhibitory effect on myeloid leukaemia cell lines by Rz1 and Rz2. Cells ($2 \times 10^4$) were incubated with 4 μg of ribozymes (Rz1 or Rz2), 0.75 μg of DOTAP and 40 U of rRNasin in 100 μl of Opti-MEM I Reduced Serum medium. After 12 h, 100 μl of RPMI 1640 medium containing 20% FBS were added to the culture medium. Then 100 μl of supernatant was replaced by an equal amount of RPMI 1640 medium containing 10% FBS, 4 μg of ribozymes, 0.375 μg of DOTAP and 20 U of rRNasin each day. Cell growth was evaluated with an MTT assay after a 5-day incubation. The results are expressed as the percentage of $OD_{570\ nm}$ in various cell lines treated with ribozymes compared with control cultured with only DOTAP in each cell line

UF-1 (established in our laboratory) (Kizaki et al, 1996) cells. The cells were all maintained in RPMI 1640 medium (GIBCO-BRL, Gaithersburg, MD) with 10% fetal bovine serum (FBS) (Cytosystems, New South Wales, Australia), 100 U $ml^{-1}$ penicillin and 100 μg $ml^{-1}$ streptomycin in a humidified atmosphere with 5% $CO_2$. All-*trans*-retinoic acid (RA) was purchased from the Sigma Chemical Co. (St. Louis, MO), and dissolved in 100% ethanol to stock concentration of 1 mM, stored at –20°C and protected from light.

### Production of ribozyme

The designs of ribozymes targeted against *AML1*/*MTG8*, Rz1 and Rz2, are illustrated in Figure 1. We also designed scramble ribozyme (ScRz) which has random ribonucleotides in 3′- and 5′-complementary arms (Figure 1). Rz1, Rz2 and ScRz were produced by in vitro transcription as described previously (Matsushita et al, 1995). Briefly, the template cDNA for Rz1 is 5′-GAG AAC CTT TCG ACC TCA CGG TCT CAT CAG GAA ATC GTA CCC TAT AGT GAG TCG TAT TAC ATG-3′, that for Rz2 is 5′-CCT CGA AAT TTC GTC CTC ACG GAC TCA TCA GGT ACT GAG CCC TAT AGT GAG TCG TAT TAC ATG-3′ and that for ScRz is 5′-NNN NNN NNT TTC GTC CTC ACG GAC TCA TCA GNN NNN NNN CCC TAT AGT GAG TCG TAT TAC ATG-3′. They were mixed with the other oligodeoxynucleotide, 5′-CAT GTA ATA CGA CTC ACT ATA GGG-3′, to form a hemiduplex, and then they were incubated at 37°C for 4 h in a 200-μl volume containing 6 mM $MgCl_2$, 2 mM each of ATP, GTP, CTP and UTP (Boehringer Mannheim, Indianapolis, IN), 240 U of rRNasin (Promega, Madison, WI, USA), 1000 U of T7 RNA polymerase and buffer for T7 RNA polymerase (New England Biolabs, Beverly, MI, USA). After incubation with 20 U of RQ1 RNase-free DNase (Promega) at 37°C for 15 min, phenol–chloroform extraction and ethanol precipitation was performed. The produced ribozyme was resuspended in DEPC-treated water and then the concentration was checked with a spectrophotometer.

### Ribozyme transfection into various leukaemic cells with lipofection

Ribozymes were transfected into various myeloid leukaemia cell lines with DOTAP (Boehringer Mannheim) for 5 days as described (Matsushita et al, 1995).

### Assays for cellular proliferation

The cells were incubated for 5 days with and without ribozymes (Rz1, Rz2 and ScRz) in a 96-well plate (Flow Laboratories, Irvine, CA, USA). Twenty microlitres of MTT (5 μg $ml^{-1}$) were added to each well. The reaction was stopped after 4 h of incubation by adding 100 μl of 0.04 N HCl in propranol and then the $OD_{570}$ was measured.

### Detection of target RNA cleavage by ribozymes

This quantification of cleavage activity by ribozymes was based on the modified method by Leopold et al (1995). Briefly, UF-1 cells ($1 \times 10^6$) which carry t(15;17) were added to various amounts ($0–5 \times 10^3$) of Kasumi-1 cells carrying t(8;21). Two hundred micrograms of ribozymes with 37.5 μg of DOTAP and 2000 U of rRNasin in 5 ml of Opti-MEM I Reduced Serum Medium (GIBCO-BRL) were added and incubated at 37°C for 12 h, and then 5 ml of RPMI 1640 medium with 20% FBS was added and incubated at 37°C for a further 12 h. The cells were then harvested, and the total RNA was isolated with Isogen (Nippongene, Toyama, Japan), and 1 μg of RNA was then applied to the reverse transcription-polymerase chain reaction (RT-PCR). RT was performed with Superscript II (GIBCO-BRL) and random primers; pd(N)6 (Takara Shuzo, Shiga, Japan) at 37°C for 60 min. PCR was performed with Taq Polymerase (Perkin-Elmer Cetus, Norwalk, CT, USA). The thermal condition of PCR was as follows: precycle at 94°C for 5 min followed by 40 cycles at 94°C for 1 min, 60°C for 1 min and 72°C for 1 min. The primers for the amplification of *AML1*/*MTG8* were 4S [5′-GAC CAT CAC TGT CTT CAC AA-3′, residue 2022 to 2041 in *AML1* (Miyoshi et al, 1993)] and 5R [5′-GTC TTC ACA TCC ACA GGT GA-3′, residue 2143 to 2162 in *MTG8* (Miyoshi et al, 1993)] which was used in our previous study (Muto et al, 1996). The β-actin gene was amplified with Human β-Actin Control Amplimer Set (Clontech, Palo Alto, CA, USA) as an internal control. The PCR products were electrophoresed in 3% agarose gel and subsequent Southern blot analysis was performed with the ECL 3′-oligolabelling and detection systems (Amersham, UK). The probe sequence for the *AML1*/*MTG8* junction site was 8P (Muto et al, 1996) [5′-CGA GAA CCT CGA AAT CGT ACT GAG A-3′, residue 2098 in *AML1* to 2122 in *MTG8* (Miyoshi et al, 1993)]. The density of the signals on the autoradiographs were analysed with Digital Densitorol DM-303 (Advantec Toyo, Tokyo, Japan).

### Detection of DNA ladder formation in Kasumi-1 cells by ribozymes

After incubation with ribozymes for 7 days, Kasumi-1 cells were washed with PBS twice and then DNA was extracted by incubating with 100 μl of buffer which includes 10 mM Tris-HCl (pH 7.4), 0.5 M EDTA (pH 8.0) and 0.5% Triton X-100 at 4°C for 10 min. After centrifugation, the supernatant was transferred into a new tube and incubated at 37°C for 1 h with 40 μg of RNase A (Sigma), and for a further hour with 40 μg of Proteinase K (Sigma). Ethanol precipitation was performed and resuspended

© Cancer Research Campaign 1999

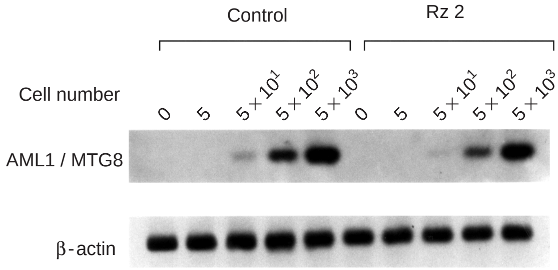

**Figure 3** Decreased expression of *AML1/MTG8* mRNA in ribozyme-treated Kasumi-1 cells. Kasumi-1 cells (0–5 $\times$ 10$^3$) were mixed with UF-1 cells (1 $\times$ 10$^6$), a myeloid leukaemia cell line without t(8;21), in 5 ml of Opti-MEM I Reduced Serum medium. The cells were transfected with 200 μg of ribozymes, 37.5 μg of DOTAP and 2000 U of rRNasin. After 12 h incubation at 37°C, 5 ml of RPMI 1640 medium with 20% FBS was added to the medium and incubated for more 12 h. Thereafter, 1 μg of RNA was applied to RT-PCR performed with Superscript II and random primers, and a subsequent Southern blot analysis were performed. Expression of *AML1/MTG8* transcript relative to β-actin in each corresponding lane was determined by densitometer

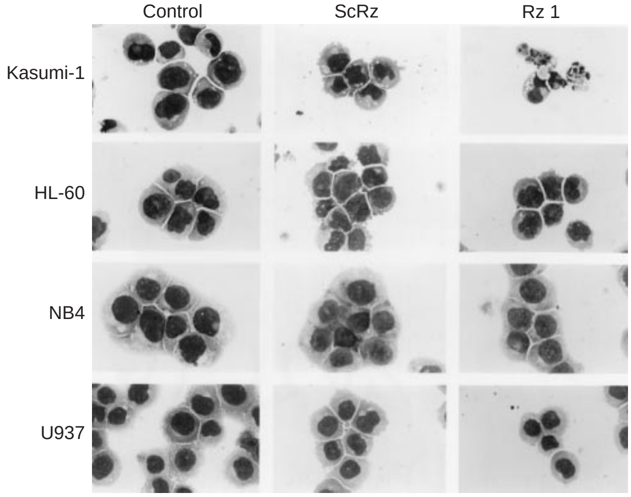

**Figure 4** Morphological changes of ribozyme-treated Kasumi-1, HL-60, NB4 and U937 cells. Cells were incubated with ribozymes (Rz1 and Rz2) and scramble ribozyme (ScRz) as a control for 7 days. Giemsa staining was performed after cytospin (original magnification, $\times$ 1000)

with TE buffer. Purified DNA was labelled with $^{32}$P-dCTP and labelled DNA was then electrophoresed in 1.8% agarose gel (Rösl, 1992). The gel was then dried on 3MM Whatman paper and thereafter autoradiography was performed.

### Assays for cellular differentiation by ribozymes

After incubation for 5 days with ribozymes, the cells were incubated for 30 min with human AB serum, and then were stained with PE-conjugated mouse anti-human CD11b antibodies and FITC-conjugated mouse anti-human CD14 antibodies (Becton Dickinson, San Jose, CA). Control studies were performed with a nonbinding control mouse IgG isotype antibody (Becton Dickinson). A flow cytometric analysis was performed with Ortho Cytoron Absolute (Ortho Diagnostic systems, Tokyo, Japan).

### RESULTS

### Cell-specific inhibition by ribozymes against AML1/MTG8

To confirm the cell-specific inhibition by ribozymes against *AML1*/*MTG8*, we transfected these ribozymes into various myeloid leukaemia cell lines, including Kasumi-1, HL-60, KG-1, U937, NB4 and UF-1 cells. Incubation with myeloid leukaemic cells and ribozymes for 5 days and then cellular proliferation was evaluated by an MTT assay. Both ribozymes only inhibited the proliferation of Kasumi-1 cells, but not the other myeloid leukaemia cell lines. Rz2 was slightly more potent than Rz1 in decreasing the absorbance of the MTT assay (Figure 2). These results suggest that Rz1 and Rz2 specifically inhibit the growth of myeloid leukaemia cells carrying t(8;21)(q22;q22).

### Cleavage activity of ribozymes in cells

To address the specific cleavage activity of ribozymes against *AML1*/*MTG8* in vitro, we next transfected Rz2 into Kasumi-1 cells diluted with UF-1 cells. UF-1 cells were used for dilution because they do not have t(8;21)(q22;q22), and were not inhibited by Rz1 and Rz2 while they also demonstrated a long doubling time (72 h)

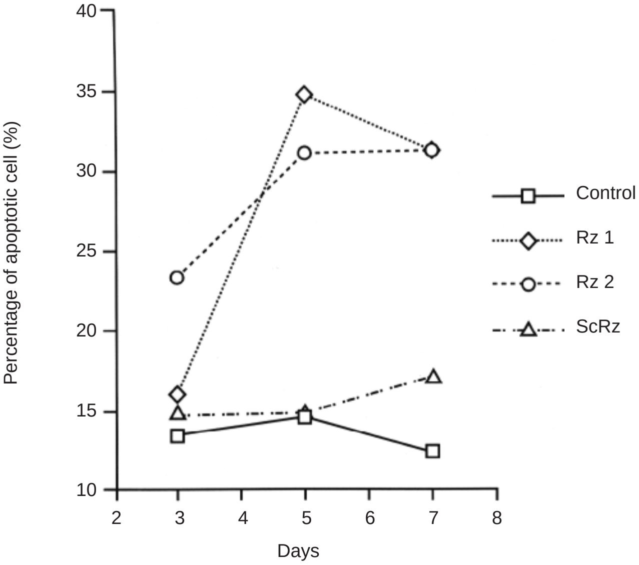

**Figure 5** Effects of ribozymes against *AML1/MTG8* chimeric transcript and ScRz on apoptosis in Kasumi-1 cells. Kasumi-1 cells were treated with ribozymes (Rz1 and Rz2), ScRz or DOTAP alone (control) up to 7 days. The percentage of cells exhibiting morphological characteristics of apoptosis was determined on cytospin slides stained with Giemsa

(Leopold et al, 1995) (Figure 2 and data not shown). Rz2 decreased the detectable level of *AML1*/*MTG8* mRNA at all corresponding lanes (Figure 3). The signal intensity was decreased to 41.1% by Rz2 in the presence of 5 $\times$ 10$^3$ Kasumi-1 cells (Figure 3). These results suggest that the growth inhibition of myeloid leukaemia cells with t(8;21)(q22;q22) by ribozymes are thus thought to be due to the reduction of *AML1*/*MTG8* mRNA.

### Detection of apoptosis in Kasumi-1 cells by ribozymes

After 7 days of culture in Rz1-, Rz2- and ScRz-transduced Kasumi-1, HL-60, NB4 and U937 cells by lipofection, morphological

 © Cancer Research Campaign 1999

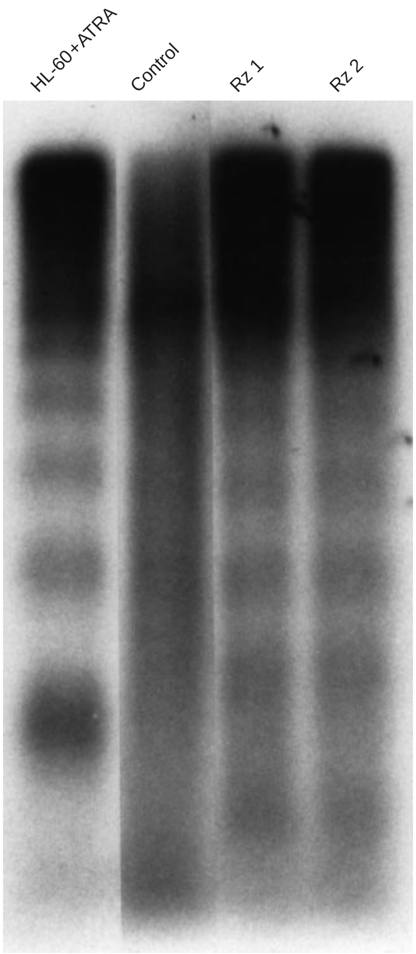

**Figure 6** Detection of DNA ladder formation in Kasumi-1 cells by ribozymes. Kasumi-1 cells were incubated with ribozymes for 5 days, after harvesting the cells. DNA was extracted and labelled with $^{32}$P-dCTP. Thereafter, it was electrophoresed in 1.8% agarose gel and then autoradiography was performed. DNA ladder formation was detected in the Kasumi-1 cells treated with ribozymes. DNA from the Kasumi-1 cells incubated without ribozymes was used as a negative control. DNA from HL-60 cells treated with all-*trans* retinoic acid was used as a positive control

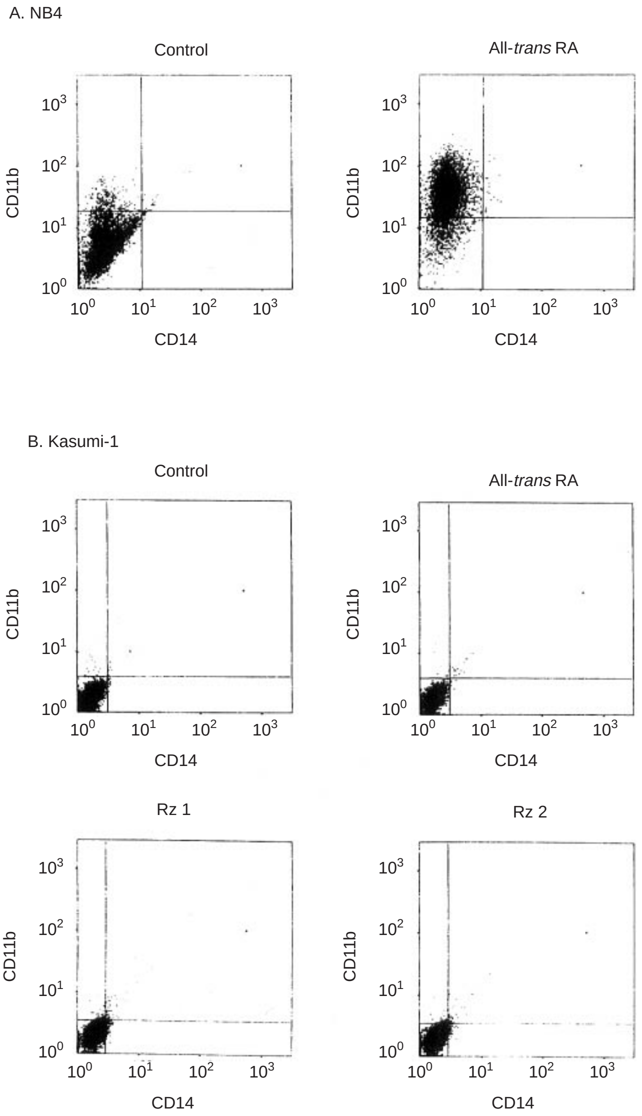

**Figure 7** Expression of CD11b and CD14 antigens by a FACS analysis. NB4 and Kasumi-1 cells were treated with all-*trans* RA ($10^{-7}$ M) for 4 days. Kasumi-1 cells were also incubated with liposome alone and ribozymes against *AML1/MTG8* (Rz1 and Rz2) for 5 days. The cells were incubated for 30 min with human AB serum to block Fc receptors and then were stained with direct immunofluorescence using FITC-conjugated mouse antihuman CD14 and PE-conjugated mouse antihuman CD11b antibodies. Control studies were performed with non-binding control mouse $IgG_1$ and $IgG_{2a}$ isotype antibodies changes of apoptosis in only Kasumi-1 cells occurred with chromatin condensation, fragmentation, and the formation of apoptotic bodies (Figure 4). However, neither Rz1 or Rz2 induced apoptosis of myeloid leukaemic cells without t(8;21). In addition, ScRz, an unrelated ribozyme, did not respond to Kasumi-1 cells (Figure 4).

The time course of the appearance of apoptotic cells during Kasumi-1 culture for 7 days was determined (Figure 5). In Rz1- and Rz2-treated Kasumi-1 cells, the percentage of apoptotic cells began to increase after day 3, and became maximal at day 5 (35% with Rz1 and 32% with Rz2). In contrast, apoptosis was not induced by the treatment of ScRz and DOTAP alone (control) in Kasumi-1 cells over a 7-day period (Figure 5). Apoptosis was also confirmed by DNA electrophoresis which showed a pattern of DNA fragments that results from the activation of endogeneous endonuclease (Figure 6). Taken together, these results indicate that the ribozymes against *AML1/MTG8* chimeric transcript specifically inhibited the proliferation of Kasumi-1 cells through the apoptotic pathway.

### Investigation of differentiation in Kasumi-1 cells by ribozymes

Induction of differention of Kasumi-1 cells into mature granulocytes or monocytes by Rz1 and Rz2 was assumed by the expression of CD11b and CD14 antigens. NB4 cells were treated with all-*trans* RA ($10^{-7}$ M) for 4 days and a cell surface marker analysis was performed as a positive control. All-*trans* RA induced differentiation of NB4 cells to mature granulocytes and increased the expression of CD11b antigen by six-fold as compared with the control cells (Figure 7 and data not shown). However, all-*trans* RA as well as Rz1 and Rz2 did not individually alter the expression of

© Cancer Research Campaign 1999

both antigens in Kasumi-1 cells by a flow cytometric analysis. In addition, no morphological changes accompanied with differentiation were observed in the Rz1-treated Kasumi-1 cells (Figure 4).

### DISCUSSION

*AML1*/*MTG8* is thought to play a key role in leukaemogenesis because the growth of leukaemic cells with t(8;21)(q22;q22) was inhibited by antisense oligonucleotides or ribozymes against *AML1*/*MTG8* fusion transcript (Sakakura et al, 1994 ;Matsushita et al, 1995; Kozu et al, 1996). The molecular mechanism of leukaemogenesis by *AML1*/*MTG8* has been studied, and AML1/MTG8 has been shown to block AML1b transcription as the dominant negative protein which is mediated by the runt homology domain (Meyers et al, 1993, 1995). More recently, it has reported that AML1 is essential for definitive haematopoiesis of all lineages by using *AML1* knockout mice (Okuda et al, 1995; Wang et al, 1996) and *AML1*/*MTG8* knock-in analyses (Yergeau et al, 1997; Okuda et al, 1998). On the other hand, few studies about the biological effect of *AML1*/*MTG8* have been reported which suggest that Kasumi-1 cells were induced to differentiate to monocytic lineage by antisense oligonucleotide complementary to the *AML1*/*MTG8* transcript, however, only 12% of the cells treated with 10 mM antisense oligonucleotide were positive for NSE staining (Sakakura et al, 1994).

We constructed two hammerhead ribozymes targeted against *AML1*/*MTG8*, and proved that these ribozymes cleaved the target substrate specifically in a cell-free system and also inhibited the growth of leukaemic cells with t(8;21)(q22;q22) (Matsushita et al, 1995). Another group has also reported similar results (Kozu et al, 1996), however, the mechanisms of the ribozymes against *AML1*/*MTG8* on the growth inhibition of myeloid leukaemic cells have yet to be elucidated. The ribozyme-induced apoptotic cells were identified based on morphology. The percentage of apoptotic cells was increased in a time-dependent manner by ribozymes targeted against *AML1*/*MTG8*. In contrast, apoptosis was not induced by the treatment of unrelated ribozyme. Therefore, the ribozyme-induced growth inhibition of Kasumi-1 cells was associated with the induction of apoptosis. We could also detect DNA ladder formation in ribozyme-treated Kasumi-1 cells. These findings suggest that the decrease in the expression of *AML1*/*MTG8* by ribozymes may thus induce leukaemic cells to apoptosis, and this is the first report which suggests that blocking the expression of *AML1*/*MTG8* by ribozymes induces apoptosis of leukaemic cells. A previous report showed that AML1/MTG8 fusion protein activates the transcription of Bcl-2 (Klampfer et al, 1996). We therefore examined the expression of Bcl-2, Bcl-Xs/L and Bax in ribozyme-treated Kasumi-1 cells by a Western blot analysis, however, the expression of these proteins was equal to those in the nontreated cells (data not shown). Further studies are thus needed to clarify the molecular mechanisms of apoptosis in ribozyme-treated leukaemic cells.

A previous study reported that the antisense oligonucleotide complementary to *AML1*/*MTG8* inhibited the growth and induced differentiation of the cell lines derived from AML containing t(8;21) (Sakakura et al, 1994). However, we could not observe any apparent morphological differentiation changes in the ribozyme-treated Kasumi-1 cells, or any apparent increase in the granulocytic lineage marker CD11b and monocytic lineage marker CD14 in these cells by a flow cytometric analysis. These data suggest that the induction of differentiation did not occur in the ribozyme-mediated growth inhibition of Kasumi-1 cells.

The transduction efficiency was evaluated by methods based on those of Leopold et al (1995) and the ribozyme decreased the level of target RNA with liposome by one-log in their study. The reduction of target RNA in our case was thus the same level as that reported in their study (Figure 3). It is possible that a more effective induction of apoptosis might also occur by improving the transduction method.

In summary, we observed both specific growth inhibition and the induction of apoptosis by inhibiting the expression of chimeric RNA by ribozymes in Kasumi-1 cells. Therefore, chimeric RNA may be useful as therapeutic targets in patients with AML. Ribozymes are thus expected to be potentially useful as specific gene modifiers for various malignancies including leukaemia (Lange et al, 1993; Shore et al, 1993; Snyder et al, 1993; Pace et al, 1994; Pachuk et al, 1994). We thus conclude that ribozymes may be useful as a new therapy based on the molecular pathogenesis in the treatment of AML with t(8;21).

### ACKNOWLEDGEMENTS

This work was supported by grants from the Ministry of Education, Science and Culture of Japan, the National Grant-in-Aid for the establishment of a High-Tech Research Center in a Private University, as well as a Keio University Special Grant.

### REFERENCES

Asou H, Tashiro S, Hamamoto K, Otsuji A, Kita K and Kamada N (1991) Establishment of a human acute myeloid leukemia cell line (Kasumi-1) with 8;21 chromosome translocation. *Blood* **77**: 2031–2036

Cameron S, Taylor DS, TePas EC, Speck NA and Mathey-Prevot B (1994) Identification of a critical regulatory site in the human interleukin-3 promoter by *in vivo* footprinting. *Blood* **83**: 2851–2859

Erickson P, Gao J, Chang K-S, Look T, Whisenant E, Raimondi S, Lasher R, Trujillo J, Rowley J and Drabkin H (1992) Identification of breakpoints in t(8;21) acute myelogenous leukemia and isolation of a fusion transcript, *AML1*/*ETO*, with similarity to Drosophila segmentation gene, runt. *Blood* **80**: 1825–1831

Frank R, Zhang J, Uchida H, Meyers S, Hiebert SW and Nimer SD (1995) The AML1/ETO fusion protein blocks transactivation of the GM-CSF promoter by AML1B. *Oncogene* **11**: 2667–2674

Haseloff J and Gerlach WL (1988) Simple RNA enzymes with new and highly specific endoribonuclease activities. *Nature* **334**: 585–591

Homann M, Tzortzakaki S, Rittner K, Sczakiel G and Tabler M (1993) Incorporation of the catalytic domain of a hammerhead ribozyme into antisense RNA enhances its inhibitory effect on the replication of human immunodeficiency virus type 1. *Nucleic Acids Res* **21**: 2809–2814

Kizaki M, Matsushita H, Takayama N, Muto A, Ueno H, Awaya N, Kawai Y, Asou H, Kamada N and Ikeda Y (1996) Establishment and characterization of a novel acute promyelocytic leukemia cell line (UF-1) with retinoic acid-resistant features. *Blood* **88**: 1824–1833

Klampfer L, Zhang J, Zelenetz AO, Uchida H and Nimer SD (1996) The AML1/ETO fusion protein activates transcription of Bcl-2. *Proc Natl Acad Sci USA* **93**: 14059–14064

Koeffler HP (1987) Syndromes of acute nonlymphocytic leukemia. *Ann Intern Med* **107**: 748–758

Koizumi M, Iwai S and Ohtsuka E (1988) Construction of a series of several self-cleaving RNA duplexes using synthetic 21-mer. *FEBS Lett* **228**: 228–230

Kozu T, Sueoka E, Okabe S, Sueoka N, Komori A and Fujiki H (1996) Designing of chimeric DNA/RNA hammerhead ribozymes to be targeted against *AML1*/*MTG8* mRNA. *J Cancer Res Clin Oncol* **122**: 254–256

Lange W, Cantin EM, Finke J and Dolken G (1993) *In vitro* and *in vivo* effects of synthetic ribozymes targeted against *BCR*/*ABL* mRNA. *Leukemia* **7**: 1786–1794

Lanotte M, Martin-Thouvenin V, Najman S, Balerini P, Valensi F and Berger R (1991) NB4, a maturation inducible cell line with t(15;17) marker isolated from human acute promyelocytic leukemia (M3). *Blood* **77**: 1080–1086

Lenny N, Meyers S and Hiebert SW (1995) Functional domains of t(8;21) fusion protein, AML-1/MTG8. *Oncogene* **11**: 1761–1769

 © Cancer Research Campaign 1999

Leopold LH, Shore SK, Newkirk TA, Reddy RMV and Reddy EP (1995) Multi-unit ribozyme-mediated cleavage of bcr-abl mRNA in myeloid leukemia. *Blood* **85**: 2162–2170

Matsushita H, Kobayashi H, Mori S, Kizaki M and Ikeda Y (1995) Ribozymes cleave the *AML1*/*MTG8* fusion transcript and inhibit proliferation of leukemic cells with t(8;21). *Biochem Biophys Res Commun* **215**: 431–437

Meyers S, Downing JR and Hiebert SW (1993) Identification of AML-1 and the (8;21) translocation protein (AML-1/ETO) as sequence-specific DNA-binding proteins: the runt homology domain is required for DNA binding and protein–protein interactions. *Mol Cell Biol* **13**: 6336–6345

Meyers S, Lenny N and Hiebert SW (1995) The t(8;21) fusion protein interferes with AML-1B-1 dependent transcriptional activation. *Mol Cell Biol* **15**: 1974–1982

Miyoshi H, Kozu T, Shimizu K, Enomoto K, Maseki N, Kaneko Y, Kamada N and Ohki M (1993) The t(8;21)translocation in acute myeloid leukemia results in production of an *AML1-MTG8* fusion transcript. *EMBO J* **12**: 2715–2721

Miyoshi H, Ohira M, Shimizu K, Mitani K, Hirai H, Imai T, Yokoyama K and Ohki M (1995) Alternative splicing and genomic structure of the *AML1* gene involved in acute myeloid leukemia. *Nucleic Acid Res* **23**: 2762–2769

Muto A, Mori S, Matsushita H, Awaya N, Ueno H, Takayama N, Okamoto S, Kizaki M and Ikeda Y (1996) Serial quantification of minimal residual disease of t(8;21) acute myelogenous leukemia with RT-competitive PCR assay. *Br J Haematol* **95**: 85–94

Nuchprayoon I, Meyers S, Scott LM, Suzow J, Hiebert S and Friedman AD (1994) PEBP2/CBF, the murine homologue of the human myeloid AML1 and PEBP2β/CBFβ proto-oncoproteins, regulates the murine myeloperoxidase and neutrophil elastase genes in immature myeloid cells. *Mol Cell Biol* **14**: 5558–5568

Okuda T, Deursen JV, Hiebert SW, Grosveld G and Downing JR (1996) AML1, the target of multiple chromosomal translocation in human leukemia, is essential for normal fetal liver hematopoiesis. *Cell* **84**: 321–330

Okuda T, Cai Z, Yang S, Lenny N, Lyu CJ, van Deursen JM, Harada H and Downing JR (1998) Expression of a knocked-in AML1-ETO leukemia gene inhibits the establishment of normal definitive hematopoiesis and directly generates dysplastic hematopoietic progenitors. *Blood* **91**: 3134–3143

Pace U, Bockman JM, Miller Jr WH, Dmitrovsky E and Goldberg AR (1994) A ribozyme which discriminates in vitro between PML/RARα, the t(15;17)-associated fusion RNA of acute promyelocytic leukemia, and PML and RARα, the transcripts from the nonrearranged alleles. *Cancer Res* **54**: 6365–6369

Pachuk CJ, Yoon K, Moelling K and Coney LR (1994) Selective cleavage of *bcr-abl* chimeric RNAs by a ribozyme targeted to non-contiguous sequences. *Nucleic Acids Res* **22**: 301–307

Rösl F (1992) A simple and rapid method for detection of apoptosis in human cells. *Nucleic Acids Res* **20**: 5243

Sakakura C, Yamaguchi-Iwai Y, Satake M, Bae SC, Takahashi A, Ogawa E, Hagiwara A, Takahashi T, Murakami A, Makino K, Nakagawa T, Kamada N and Ito Y (1994) Growth inhibition and induction of differentiation of t(8;21) acute myeloid leukemia cells by the DNA-binding domain of PEBP2 and the *AML1*/*MTG8(ETO)*-specific antisense oligonucleotide. *Proc Natl Acad Sci USA* **91**: 11723–11727

Schiffer CA, Lee ED, Tomiyasu T, Wiernik PH and Testa JR (1989) Prognostic impact of cytogenetic abnormalities in patients with de novo acute nonlymphocytic leukemia. *Blood* **73**: 263–270

Shore SK, Nabissa PM and Reddy EP (1993) Ribozyme-mediated cleavage of the BCRABL oncogene transcript: *in vitro* cleavage of RNA and *in vivo* loss of P210 protein-kinase activity. *Oncogene* **8**: 3183–3188

Snyder DS, Wu Y, Wang JL, Rossi JJ, Swiderski P, Kaplan BE and Forman SJ (1993) Ribozyme-mediated inhibition of *bcr-abl* gene expression in a Philadelphia chromosome-positive cell line. *Blood* **82**: 600–605

Takahashi A, Satake M, Yamaguchi-Iwai Y, Bae SC, Lu J, Maruyama M, Zhang YW, Oka H, Arai N, Arai K and Ito Y (1995) Positive and negative regulation of granulocyte-macrophage colony-stimulating factor promoter activity by *AML1*-related transcription factor, PEBP2. *Blood* **86**: 607–616

Tanaka T, Tanaka K, Ogawa S, Kurokawa M, Mitani K, Nishida J, Shibata Y, Yazaki Y and Hirai H (1995) An acute myeloid leukemia gene, *AML1*, regulates hematopoietic myeloid cell differentiation and transcriptional activation antagonistically by two alternative spliced forms. *EMBO* **14**: 341–350

Tanaka T, Kurokawa M, Ueki K, Tanaka K, Imai Y, Mitani K, Okazaki K, Sagata N, Yazaki Y, Shibata Y, Kadowaki T and Hirai H (1996) The extracellular signal-regulated kinase pathway phosphorylates AML1, an acute myeloid leukemia gene product, and potentially regulates its transactivation ability. *Mol Cell Biol* **16**: 3967–3979

Tashiro S, Kyo T, Tanaka K, Oguma N, Hashimoto T, Dohy H and Kamada N (1992) The prognostic value of cytogenetic analysis in patients with acute nonlymphocytic leukemia treated with the same intensive chemotherapy. *Cancer* **70**: 2809–2815

Wang Q, Stacy T, Binder M, Marin-Padilla M, Sharpe AH and Speck NA (1996) Disruption of the *cbfa2* gene causes necrosis and hemorrhaging in the central nervous system and blocks definitive hematopoiesis. *Proc Natl Acad Sci USA* **93**: 3444–3449

Yergeau DA, Hetherington CJ, Wang Q, Zhang P, Sharpe AH, Binder M, Martin-Padilla M, Tener DG and Speck NA (1997) Embryonic lethality and impairment of haematopoiesis in mice heterozygous for an AML-ETO fusion gene. *Nat Genet* **15**: 303–306

Zhang D, Fujioka K, Hetherington CJ, Shapiro LH, Chen H, Look T and Tenen DG (1994) Identification of a region which directs the monocytic activity of the colony-stimulating factor 1 (macrophage colony-stimulating factor) receptor promoter and binds PEBP2/CBF (AML1). *Mol Cell Biol* **14**: 8085–8089

Zhang D, Hetherington CJ, Meyers S, Rhoades KL, Larson CJ, Chen H, Hiebert SW and Tenen DG (1996) CCAAT enhancer-binding protein (C/EBP) and AML1 (CBFα2) synergistically activate the macrophage colony-stimulating factor receptor promoter. *Mol Cell Biol* **16**: 1231–1240

*© Cancer Research Campaign 1999*
